# A national survey of rheumatology telephone advice line support in the United Kingdom: frontline perspectives

**DOI:** 10.1093/rap/rkae084

**Published:** 2024-07-16

**Authors:** Sarah Ryan, Samantha Hider, Jay Tavernor, Andrew Hassell

**Affiliations:** Haywood Academic Rheumatology Centre, Midlands Partnership University NHS Foundation Trust, Haywood Hospital, Stoke on Trent, UK; Department of Research and Innovation, Midlands Partnership University NHS Foundation Trust, St George’s Hospital, Stafford, UK; Haywood Academic Rheumatology Centre, Midlands Partnership University NHS Foundation Trust, Haywood Hospital, Stoke on Trent, UK; Faculty of Medicine and Health Sciences, School of Medicine, Keele University, Staffordshire, UK; Department of Research and Innovation, Midlands Partnership University NHS Foundation Trust, St George’s Hospital, Stafford, UK; Haywood Academic Rheumatology Centre, Midlands Partnership University NHS Foundation Trust, Haywood Hospital, Stoke on Trent, UK; Faculty of Medicine and Health Sciences, School of Medicine, Keele University, Staffordshire, UK

**Keywords:** rheumatoid arthritis, telephone advice lines, survey

## Abstract

**Objectives:**

Telephone advice lines are a key component of rheumatology services. A national survey of telephone advice line providers was undertaken to explore how this service is currently delivered and the impact on those delivering it to inform providers, policymakers and patients.

**Methods:**

We conducted an online survey between March and September 2023 collecting data on demographics, how advice lines function, governance and the impact on nurses’ well-being. Data were analysed using descriptive statistics.

**Results:**

A total of 123 health professionals completed the survey. The majority were rheumatology nurses [*n* = 118 (96%)], >45 years of age [*n* = 112 (91%)], band ≥7 [*n* = 92 (76%)], with 77 (65%) reporting >10 years of experience within rheumatology. Most advice lines operated weekdays only [*n* = 93 (79%)], with most calls returned within 2 days [*n* = 81 (66%)], although some callers waited >7 days [*n* = 19 (15%)]. The number of calls received monthly ranged from 100 to >800, with 46 (37%) responders reporting >500 calls/month. The most common reasons for contacting advice lines were disease activity, pain and medication concerns. For most responders, governance arrangements were unclear [*n* = 72 (61%)]. Providing advice lines impacted on the well-being of nurses providing the service: 89 (72%) felt anxious ‘sometimes to mostly’ and 79 (64%) found it ‘mostly–always’ stressful. A total of 85 (69%) nurses had not received any training to manage advice lines.

**Conclusion:**

Although telephone advice lines are provided by experienced rheumatology nurses, high demand is impacting on well-being. Having designated training could equip nurses with additional skills to manage increased capacity and monitor their own well-being.

Key messages

- The demand for rheumatology telephone advice lines in the UK has increased.
- The majority of rheumatology nurses receive no training in providing telephone advice.
- Providing advice line support causes feelings of stress and anxiety among rheumatology nurses.

## Introduction

Telephone advice lines are a core feature of rheumatology services and an essential resource for providing clinical advice, support and continuity of care [1] and are highly valued by patients [2]. Some advice lines offer support and advice for patients with a range of rheumatological, usually long-term, conditions including inflammatory arthritis and connective tissue disorders, while others are more focused on the management of specific conditions such as osteoporosis. Telephone advice line services enable patients to access advice about symptoms and treatments and raise concerns about their condition, with a recommended response time of 1–2 days [3, 4]. A key component of patient self-management is the ability to readily access information from healthcare professionals to support coping strategies and care escalation as required [1].

The demand for telephone advice lines and the negative impact on the well-being of rheumatology nurses was identified prior to the COVID-19 pandemic [1] and demand has since increased further. We previously reported a tripling in patients accessing our service [5]. The demand is likely to remain high in the UK due to alterations in the delivery of rheumatology care, with an increase in remote services [6]. This poses challenges for rheumatology teams providing these services. Potential solutions for improvements in service delivery and to support staff providing these services could include specific training in conducting telephone consultations, protocols in place including how to seek clinical guidance and ensuring there is sufficient time within job plans to provide the service.

Although there is evidence of the expansion of advice line services both inside and outside of rheumatology, systematic information regarding the provision of such services in rheumatology is scant. Information regarding models of advice line provision, manning of the service, standard protocols, audits and whether the service is required by commissioning funders is generally not available. At the same time, despite recognition of the complex skills required to provide telephone advice and support [7], little is known about the resources, training, preparation and ongoing supervision clinicians receive and require to deliver this care [8]. The aim of this research was to identify how telephone advice line services are currently delivered in the UK and the impact on staff providing this service. We believe this is the first such UK rheumatology survey and hope it will provide valuable insights into how telephone advice lines are delivered and the impact on staff who provide them. This information can be used to inform service improvements in this area, including support and training for those delivering the service, aiming to improve the well-being of both patients and nurses.

## Methods

An online questionnaire developed and piloted by five rheumatology nurses with input from patients and stakeholders was advertised through the Royal College of Nursing Rheumatology Forum, the BSR and the Midlands Rheumatology Society. The survey was aimed at nurses and allied health professionals providing telephone advice line support to people with a rheumatological condition. Since no national registers of rheumatology nurses exist, it was not possible to determine the sample size for this survey.

Potential participants accessed an electronic link to an invitation letter and a questionnaire. All participants provided informed consent. As an incentive to complete the survey, respondents were offered a certificate towards revalidation.

### Data collection

Data were collected via an electronic questionnaire platform, Health Survey. The survey consisted of six sections, including demographic data of health professionals providing the service, how telephone advice lines operate, reasons for people contacting the service, documentation recorded, governance and evaluation and impact on provider well-being, using closed and free-text questions (Supplementary Data S1, available at *Rheumatology Advances in Practice* online). The survey was available from March to September 2023.

### Data analysis

Descriptive statistics were used to present the quantitative data. The free-text comments were read and coded thematically by members of the research team. Connected themes were then clustered together and further refined until the main themes were agreed upon.

### Patient and public involvement

A stakeholder group consisting of 10 members, including rheumatology nurses, rheumatologists, a service manager and people with arthritis who use telephone advice line services, was formed. The first stakeholder meeting identified current challenges in providing and accessing advice line services to inform the content of the survey questionnaire. Following the design of the questionnaire, the stakeholder group met again to discuss the format, face and content validity of the questionnaire. The duration of each stakeholder group meeting was 60–90 min.

## Results

A total of 123 people completed the survey. Judging by the 83 respondents who reported the geographic region of their service, there was good representation from across the UK (Supplementary Fig. S1*,* available at *Rheumatology Advances in Practice* online).

Demographics are described in Table 1. The majority of respondents were rheumatology nurses [*n* = 118 (96%)], >45 years of age [*n* = 112 (91%)], band ≥7 [*n* = 92 (76%)], with >10 years of experience in rheumatology [*n* = 77 (65%)], with a first degree or higher [*n* = 106 (90%)]. Forty-five (38%) nurses reported a prescribing qualification.

**Table 1.** Respondent characteristics

Profession	Rheumatology nurses, *n* = 118 (96%); pharmacist, *n* = 1; matron, *n* = 1; senior nurse, *n* = 1; other, *n* = 2
Age (years)	35–44, *n* = 8 (6%)
	45–54, *n* = 55 (45%)
	54–46, *n* = 57 (46%)
	Prefer not to say, *n* = 3 (2%)
Gender	Female, *n* = 114 (93%); male, *n* = 9 (7%)
Banding	6, *n* = 30 (24%)
	7, *n* = 70 (57%)
	8, *n* = 21 (17%)
	Other, *n* = 2 (1%)
Ethnicity	Female white British, *n* = 105 (85%)
	Female from other ethnic groups including Asian or Asian British, Black, Black British, Caribbean or African, *n* = 8 (6%)
	Female other than white, white European, mixed or mixed ethnic groups, white mixed, *n* = 7 (5%)
	Male white British, Irish or other, *n* = 3 (2%)
Years in rheumatology	<1, *n* = 1
	1–3, *n* = 9 (7%)
	4–6, *n* = 19 (15%)
	7–9, *n* = 17 (14%)
	>10, *n* = 77 (63%)
Education	MSc, *n* = 40 (32%)
	BSc, *n* = 64 (52%)
	Diploma, *n* = 42 (34%)
	Non-medical prescriber, *n* = 45 (36%)
	Post-graduate certificate in advanced nursing, *n* = 1

The majority of telephone advice lines provided an initial automated response [*n* = 90 (76%). Recorded calls were then usually triaged [*n* = 79 (64%)] by clinical support workers/administrators [*n* = 44 (37%)] and/or rheumatology nurses [*n* = 29 (25%)]. The number of calls received over 1 month ranged from 100 to >800, with 46 (37%) responders reporting >500 calls/month. The majority of respondents reported a service in which 8 h/day, 5 days/week, were spent responding to advice line calls, with calls returned within 2 days [*n* = 81 (66%)] by one to two rheumatology nurses [*n* = 72 (61%)], although some calls took >7 days for a response [*n* = 19 (15%)] (Table 2). Use of a triage system did not always lead to a response time within 2 days: in 37 cases it did, in 27 cases it did not, compared with 34/53 cases reporting a response time within 2 days in those who did not use a triage service. The main reasons for contacting telephone advice lines included increased disease activity, managing pain and concerns relating to DMARDs.

**Table 2.** How telephone advice line services function

Function		*n*
Over what time period all calls are responded to	<4 h/day	5
	4 h/day	15
	6 h/day	23
	8 h/day	68
	Other	6
Is there a system for prioritising calls?	Yes	75
	No	48
Who prioritises calls?	Rheumatology nurses	29
	Clinical support worker	23
	Administrator	21
	Other	6
Typical response time	<24 h	44
	1–2 days	37
	3–4 days	14
	5–6 days	8
	>7 days	19
	Other (dependent on staffing levels)	1
Who is involved in giving advice?	Rheumatology nurse	118
	Pharmacist	17
	Doctor	1
	Other	5
How many health professionals provide advice on a daily basis?	1	34
	2	38
	3	21
	>3	22
	Other	8
How many calls are received within a typical month?	<100	4
	100–300	20
	300–500	40
	700–800	3
	>800	15
	Other	1
	Don’t know	2

Telephone advice line services were reported as being commissioned by 37 (30%) respondents, as not being commissioned by 54 (44%), with 32 (26%) unaware as to whether or not they were commissioned. In 25/30 cases of commissioned service, response times were reported as being within 2 days, compared with 48/93 cases who did not report a commissioned service.

The majority of telephone advice line services did not have standard operating procedures (SOPs) [*n* = 72 (61%)] and most did not have provision for people whose first language is not English [*n* = 99 (84%)]. Sixty-seven respondents (82%) reported that all calls were documented in the patient’s clinical record. Just over half of respondents [*n* = 67 (54%)] reported that the advice line service was audited based on local criteria that included the nature of the advice requested, response given, time taken to return the call and duration of the call (Table 3).

**Table 3.** Governance and evaluation

Governance		*n*
Manned or answerphone service	Answerphone	90
	Manned	33
Commissioned service	No	54
	Yes	37
	Don’t know	32
Provision for people whose first language is not English	No	99
	Yes	24
SOPs	No	72
	Yes	51
Telephone advice line data collected	Yes	82
	No	41
	Nature of advice requested	74
	Response given	57
	Time taken to return call	47
	Duration of call	38
Call documented	Yes	120
	On the clinical record	101
	On an audit form	22
	No	3
Is the system audited?	Yes	67
	No	56
Availability (days/week)	<4	4
	4	12
	5	93
	7	13
	Other	1

Providing telephone advice line services was reported to negatively impact provider well-being; 89 (75%) felt anxious ‘sometimes to mostly’ and 79 (67%) found it ‘mostly–always’ stressful. Reported stress did not appear related to years of experience: 60% of respondents with >10 years of experience (*n* = 68) reported providing advice lines as ‘always’ or ‘mostly’ stressful, compared with 67% of those with <6 years of experience (*n* = 30). A total of 85 (72%) providers had not received any training to support managing the advice line and 79 (67%) reported that cover was often not provided for leave or sickness.

The free-text comments (Supplementary Data S2, available at *Rheumatology Advances in Practice* online) identified three main themes:

### Theme 1: the stress experienced by nurses providing telephone advice

The majority of nurses found providing telephone advice line services stressful due to a lack of training, patients’ expectations, the complexity of questions and the requirement to return calls in a timely manner.

The advice line is routinely seen as being the single most stressful aspect of the specialist rheumatology nursing role but has the least support in terms of training, time to respond to patients and complexity of questions. — Respondent 22

Providing advice over the telephone can be challenging and stressful when patients’ expectations are not met. — Respondent 58

### Theme 2: redesigning the service

Service improvements included having designated appointment times and utilising the skills of other team members to triage calls.

Our calls are booked into 15-min slots by an administrator, and we always know exactly when we are working on the advice line and the number of patients we need to call. This has reduced stress levels enormously. — Respondent 28

Health care assistant triage has been a revelation; they filter out a lot of urgent calls and medical queries they put straight through to consultant. — Respondent 73

### Theme 3: inappropriate telephone advice line use

Respondents identified that many calls received reflected the challenges patients faced accessing general practitioner (GP) services or resulted from the cancellation of rheumatology appointments.

Patients abuse the advice line as they struggle to get GP appointments, so leave messages about non-rheumatology issues. This clogs up the system, thus delays replies to genuine rheumatology patients. — Respondent 47

We find that patients are increasingly using the advice line service as a replacement clinic service as their clinic appointments are frequently cancelled. — Respondent 106

## Discussion

This is the first national survey of rheumatology telephone advice line use and highlights the increased demand for such services and the negative impact that this can have on an already stretched rheumatology nursing workforce [1]. Key findings were that telephone advice lines are predominantly staffed by experienced rheumatology nurses via an automated message answerphone service, 5 days/week, with the majority of calls triaged and responded to within the national guidance of 1–2 days [4, 9]. Rheumatology nurses had not received any standardised training to provide telephone advice and found this aspect of their role stressful and anxiety producing. Many felt that current advice line services were not sustainable and needed to be redesigned and a process implemented for redirecting inappropriate callers. This could involve making the purpose of advice lines clearer to users and highlighting situations where it would be more appropriate to contact other services such as primary care or pharmacy.

The literature indicates that advice lines provide an important part of the management and support of people with inflammatory arthritis and other chronic diseases and are for providing clinical advice, reassurance and continuity of care [1, 2]. People reported increased confidence in their ability to self-manage their rheumatology condition when they had rapid access to health professionals with specialist knowledge and were less likely to seek other healthcare resources [10–11]. Advice line services that promote self-management can contribute to better treatment and health outcomes by addressing pain, fatigue and well-being, and people with arthritis have expressed a desire to engage with technology that supports self-management [12, 13].

National and European guidelines for the management of inflammatory conditions [3, 7, 9] endorse the need for rapid access to specialist care to manage increased disease activity and medication side effects, with a target of 1–2 days to respond to calls. Our survey demonstrated that despite an increase in the volume of calls, the majority of respondents felt that their rheumatology department was still achieving the target for response times, although the pressure of dealing with this increased workload was impacting negatively on the psychological well-being of rheumatology nurses providing this service.

Several rheumatology services within the UK have redesigned how telephone advice lines operate in response to the increased demand. This has included using computerised systems to provide specific appointment times for calls to be returned, ensuring there are two nurses at any one time returning calls to provide peer support and making clear the purpose of advice lines to try and reduce the number of calls not directly related to rheumatology conditions [14–16].

Our findings identified that the majority of telephone advice lines did not have SOPs. The advantage of having a SOPs is to ensure consistent, safe management, clear algorithms for escalation, to allow for clear governance and to identify any omissions in service provision.

The responses to the question regarding commissioning are a little difficult to interpret. National Health Service commissioning differs in the UK, with arrangements changing over the last few years. It is possible that respondents would be unaware of commissioning arrangements. Nevertheless, it is important, because if funders require specific identified services, they are more likely to be provided and sustainably resourced. The content of some free-text responses suggests that this aspect of the rheumatology service has proliferated in the absence of clear planning and resourcing.

Nurses within our survey were experiencing stress and anxiety, which concurs with the findings of an earlier survey into the increasing demands being experienced by rheumatology clinical nurse specialists in all aspects of their role. In a survey of 158 rheumatology nurse specialists in the UK undertaken pre-pandemic, 83% identified that they were unable to respond to all telephone advice line calls due to workload issues, which resulted in feeling ‘overwhelmed’ or ‘stressed’ [1]. Nurses providing health-related telephone advice for all conditions (not specific to rheumatology) described feeling ‘moral distress’, as they were unable to care for callers in the way that they would want to due to the volume of calls received and were unable to meet callers’ expectations [17, 18].

The free-text comments in our survey provided insights into some of the reasons for the negative impact on well-being, with a high volume of calls and minimal staffing making current services unsustainable. An absence of support from managers and limited administrative assistance increased the pressure on rheumatology nurses. It is possible that this reflects some of the commissioning issues alluded to above. There was also recognition that patients contacting the service have complex needs and, with the lack of time available to respond to calls, it was not always possible to meet patient expectations.

In studies outside rheumatology, including those of nurses providing telephone advice line support to people with medical and mental health issues, it has been found that the occupational stress encountered by nurses often arises from a combination of high demands, cognitive fatigue, low control over workload and a lack of appreciation from managers [19, 20]. Stress can have negative consequences when demands on the individual exceed their adaptive capabilities [21]. Being able to exert a level of control over the workload and having designated recovery times from constant emotional giving can offer some protection against feeling stressed [20, 21]. Other studies suggest that having time set aside for inter- and intraprofessional discussions and feedback [18] can reduce the occupational stress of providing advice line services.

Some respondents in our survey suggested an association between increased advice line demand and the struggle to retain rheumatology nurses. High levels of stress reported by nurses providing similar services resulted in increased ill health and sick leave [21]. Addressing work-related stress is important to improve nurse well-being and job satisfaction and prevent burnout, as well as improving patient safety and outcomes of care [20]. Our respondents reported that no additional staffing was provided to cover advice line services during annual leave or sickness.

The majority of responders in our survey had not received any formal training and had learned how to provide telephone advice by observing their peers. Telephone consultation skills are part of a wider set of remote consulting skills that include actively listening to the patient, clarifying their concerns and agreeing on a plan of action [8]. Self-awareness and emotional intelligence, the ability to monitor and regulate one’s own feelings and those of others, are required to provide effective telephone advice [8, 22], especially when it is not possible to see the patient or interpret non-verbal cues and body language [23].

Specific training to provide telephone advice and support may help to recognise and manage the signs of stress as well as teaching stress reduction techniques [21]. Empathy training could be a useful component, as telephone consultations have a higher risk of objectifying the person than occurs in face-to-face consultations [21]. The involvement of decision-making tools could help nurses differentiate between minor and serious problems and provide some standardisation that could positively impact stress levels [18]. Also, skills to deal with challenging patients, frequent callers and how to manage patients’ expectations would provide nurses with different ways of addressing these potentially stressful situations [18]. A Cochrane systematic review identified the need for further research assessing the effects of different training interventions on clinicians’ consultation skills and their effect on patient outcome [8].

Alongside designated training, other solutions to optimise the effectiveness of telephone advice line service could include reviewing rheumatology nurses’ current job plans to ensure sufficient time is allocated to providing the service, utilising the skills of other team members to triage calls, optimising support services (including IT), regular review of the service, a robust governance framework and offering patients appointment times.

The strength of this research is that it is the first national survey of telephone advice line use and includes representation from across the UK. Stakeholder involvement, including patients and health professionals, was used in designing the questionnaire, thus increasing face and content validity. One limitation is that although there was wide geographical representation, we do not know exactly how many rheumatology units were represented, as there may have been more than one respondent from larger departments. Our response of 123 individuals is comparable to other surveys carried out by the Royal College of Nursing Rheumatology Forum and BSR, although since no national registers of rheumatology nurses exist, it is not possible to accurately quantify the survey response rate.

There is also a potential for selection bias, as nurses were recruited from professional organisations and the incentive of a revalidation certificate may have attracted more nurses who are close to revalidation deadlines, although this is unlikely to have affected the findings.

In the future, artificial intelligence (AI) could be used to support telephone advice line services. AI refers to computer technologies that emulate mechanisms supported by human intelligence. It is already being used in remote monitoring of patients with chronic conditions and has been well received by patients [24, 25]. It is possible that virtual assistants and clinical decision-making support via a system of algorithms [24] could be used to address some of the main reasons for contacting advice lines, including symptom management, increased disease activity and concerns regarding medications. However, while AI could be a potentially useful solution, implementing such approaches is unlikely to provide a solution for all, given the challenges of access to and confidence in digital technology and the risk of exacerbating existing health inequalities.

## Conclusion

Although telephone advice lines are staffed by experienced rheumatology nurses, constant high demand is impacting on their well-being. Patients contacting the service have complex needs, and with increasing demand, it is not always possible to meet patient expectations. Having designated training could equip nurses with additional skills to manage increased capacity and support and monitor their own well-being. Advice lines need to be appropriately resourced and regularly evaluated to ensure they meet the needs of people with arthritis without negatively impacting on provider well-being.

## Supplementary Material

rkae084_Supplementary_Data

## Data Availability

The data underlying this article are available in the article.
