# Supplementary material for: A national survey of rheumatology telephone advice line support in the United Kingdom: frontline perspectives
Source: Rheumatol Adv Pract. 2024 Jul 16;8(3):rkae084. doi: 10.1093/rap/rkae084 (PMC11272169; doi:10.1093/rap/rkae084)
Supplement: rkae084_Supplementary_Data [file rkae084_supplementary_data.docx]

**Supplementary Data S1: The Questionnaire.**

1. **Demographic Data**

Profession, age, gender, ethnicity, banding, years of experience and education.

1. **Telephone Advice Lines**
2. Does your service provide a telephone advice line service? Yes/No.
3. How many calls does the telephone advice line receive in a typical month? (Tick box option).
4. Is the telephone advice line a commissioned service? Yes/no.
5. What is the size of the population your service serves? (Tick box option).
6. Is there a system for prioritising calls? Yes/No. If Yes, who prioritises the calls. (Tick box option).
7. Is the telephone advice line service manned or answer phone? (Please circle).
8. Who is involved in giving advice? (Tick box option).
9. How many health professionals provide advice on a daily basis? (Tick box options)
10. How many days is the advice line available?
11. Over what time period are calls responded to? (Tick box options).
12. What is the target for returning calls/emails? (Tick box options).
13. In practice, what is the typical response time? (Tick box options).
14. Which of the following health professionals contact the advice line. (Tick box options/free text comment).
15. What is the percentage of patients versus health professionals that use the advice line? (Tick box option).
16. Is there any provision for people whose first language is not English? Yes/No.
17. Is there a standard operating procedure for the advice line? Yes/No.
18. Are any telephone advice line data collected? Yes/No. If Yes, please select which aspects are collected. (Tick box options).
19. Is the call documented? Yes/No. If Yes, how is it documented? (Tick box options).
20. Is the system audited? Yes/No.
21. How are people made aware of advice line services? (Tick box options).
22. **Reasons for Contacting the Advice Services**
23. Commonest reasons for using the advice services. (Tick box option).
24. Commonest responses given. (Tick box options).
25. **Support**
26. Is there designated time within your role to provide advice line support? Yes/No. If Yes, how much time per week? (Tick box options).
27. How easy is it to access medical support if it is required? (Tick box option).
28. Have you received any training to deliver advice line support? Yes/No. (Tick box option).
29. Do you feel you have unmet training needed for providing advice line services? Yes/No. If Yes, a free text section was provided for respondents to describe their training needs.
30. Do you feel you have enough support to provide an advice line service? (Options: sometimes, mostly, always, never).
31. Is providing an advice line service stressful? (Options: sometimes, mostly, always, never).
32. Does providing advice line support cause you to feel anxious? (Options: sometimes, mostly, always, never).
33. Is there cover for the advice line in the event of annual leave or sickness? (Options: sometimes, mostly, always, never).
34. **Wellbeing**
35. Do you find you have enough support to provide an advice line service? (Always, mostly, sometimes, never).
36. Is providing an advice line stressful? (Always, mostly, sometimes, never).
37. Does providing advice line support cause you to feel anxious? (Always, mostly, sometimes, never).
38. Do you worry about the amount of calls you have to respond to? (Always, mostly, sometimes, never).
39. **Ethnicity and Diversity**
40. Is data collected on who accesses the advice line service? Yes/No.
41. Is data collected on sex?
42. Is data collected on age?
43. Is data collected on ethnicity?
44. How are people made aware of the service? (Tick box option).
45. **Comments**

If you have any other comments about advice services, please add them below.

**Supplementary Figure S1: Geographical Location of Service Responders.**

**
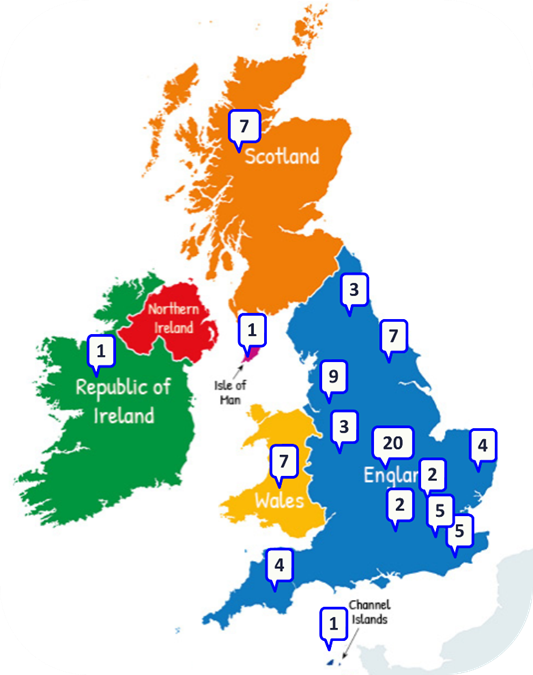
**

Key: The numbers indicate the amount of respondents in different geographical locations.

**Supplementary Data S2: Free Text Comments.**

**Three main themes: Providing an advice line is stressful, redesigning the service and inappropriate use of the service.**

1. **Providing an Advice Line is Stressful**

“The advice line is routinely seen as being the single most stressful aspect of the specialist rheumatology nursing role but has the least support in terms of training, time to respond to patients and complexity of questions.” *(Respondent 22).*

“Nurses in the past who have left the team have stated that the excessive stress and anxiety induced by the advice line has been a driving factor in them leaving.” *(Respondent 96).*

“Providing advice over the telephone can be challenging when patients expectations are not met. The can be stressful.” *(Respondent 58).*

“The advice line is the hardest part of the job. It can be overwhelming at times. I have never had any training and I have been a Rheumatology Nurse for over 20 years. The complexity of the calls is hard to manage sometimes.” *(Respondent 100).*

“The nurse team is stretched beyond capacity and the advice line is such a huge workload that it means we all spend many extra hours a week (unpaid) to ensure calls are returned.” *(Respondent 120).*

“Answering the calls full time is unfair on clinicians, it is too much emotional.” *(Respondent 124).*

1. **Redesigning the Service**

“We conducted a re-design of our advice line service just after the pandemic started. Our calls are booked into 15-minute slots by an administrator, and we always know exactly when we are working on the advice line and the number of patients we need to call. This has reduced stress levels enormously.” *(Respondent 28).*

“We have quite a good set up with call triage and downloading and we have trained B4 data coordinator to download and flag urgent calls. We (nurses) also share the calls and are quite flexible depending on other clinical work.” *(Respondent 45).*

“We have made a lot of changes to our advice line since Covid. Health care assistant triage has been a revelation; they filter out a lot of urgent calls and medical query they put straight through to Consultant.” *(Respondent 73).*

“The advice line is under review after a new part-time nurse joined the team and has been looking at everything with 'fresh eyes'. We may change to set times/days for responding to calls/e-mails rather than responding adhoc.” *(Respondent 76).*

“Advice is integral to the CNS role - difficult to define and should be a commissioned service.” *(Respondent 56).*

“We are hoping to keep the answerphone facility but have a patient self-booking service online (like booking a gym class). The admin support spends 50% of her full working week listening to calls, filtering to appropriate channels and booking the call in.” *(Respondent 8).*

“Managers are currently conducting audits on the service and new methods of working such as named allocation of staff taking calls in blocks of 2 hours.” *(Respondent 32).*

1. **Inappropriate Use of the Service**

“Patients abuse the advice line as they struggle to get GP appointments, so leave messages about non rheumatology issues, this clogs up the system, thus delays replies to genuine rheumatology patients.” *(Respondent 47).*

“We have seen an increasing use of the advice line year on year which has escalated further as patients find it more difficult to access their own GP services.” *(Respondent 63).*

“There has been an increase of patients contacting advice line with issues they would have previously contacted GP practice for advice (pre-Covid).” *(Respondent 80).*

“We find that patients are increasingly using the advice line service as a replacement clinic service as their clinic appointments are frequently cancelled.” *(Respondent 106).*

“Frequently it feels like a conveyer belt service and staff are often unable to provide psychological support to patients. One of the problems we are trying to address is patients who frequently call the advice line.” *(Respondent 62).*
